# Pectin enhances the effect of fecal microbiota transplantation in ulcerative colitis by delaying the loss of diversity of gut flora

**DOI:** 10.1186/s12866-016-0869-2

**Published:** 2016-11-03

**Authors:** Yao Wei, Jianfeng Gong, Weiming Zhu, Hongliang Tian, Chao Ding, Lili Gu, Ning Li, Jieshou Li

**Affiliations:** Department of General Surgery, Jinling Hospital, Medical School of Nanjing University, 305 East Zhongshan Road, Nanjing, 21002 China

**Keywords:** Fecal microbiota transplantation, Ulcerative colitis, Pectin, Mayo score, Diversity

## Abstract

**Background:**

Fecal microbiota transplantation (FMT) induces remission in ulcerative colitis (UC). However, the treatment effect of FMT diminishes over time. Maintaining the diversity of the gut flora for long periods may improve the effects of FMT in UC. Pectin, which can be fermented by gut microbiota into short-chain fatty acids, is postulated to shape the composition and maintain the balance of gut microbiota following transplantation. This study investigated whether pectin could enhance the effects of FMT in UC patients.

**Results:**

Three FMT patients and four FMTP patients achieved the primary outcome. The Mayo scores of the FMTP group were lower than those of the FMT group at weeks 4 and 12 (*P* = 0.042 and *P* = 0.042, respectively). There were no differences in the diversity of the gut flora between the two groups at weeks 4 and 12; however, the composition of the gut flora of the FMTP group was more similar than the FMT group to that of the donor at all-time points post-treatment.

**Conclusions:**

Pectin decreased the Mayo score by preserving the diversity of the gut flora following FMT for UC.

**Trial registration:**

Current Controlled Trial NCT02016469. Registered 10 November 2013

**Electronic supplementary material:**

The online version of this article (doi:10.1186/s12866-016-0869-2) contains supplementary material, which is available to authorized users.

## Background

Ulcerative colitis (UC) is a lifelong disease caused by an interaction between genetic and environmental factors. The precise etiology is unknown, and therapeutic options are limited. Fecal microbiota transplantation (FMT), infusion of a fecal preparation from a healthy donor into the gastrointestinal (GI) tract of a patient, has been trialed as a therapeutic approach to diseases associated with dysbiosis of the gut [1]. FMT is an effective therapy for recurrent *Clostridium difficile* infection [2, 3], but outcomes of FMT in patients with other GI diseases, such as UC, have been varied [4–7]. Most studies of UC have suggested that FMT initially produces beneficial effects, but these are diminished over time.

Apple-derived pectin is a soluble fiber. It can be fermented by gut microbiota in the colon into short-chain fatty acids (SCFA). Pectin may also help maintain the balance of gut microbiota [8]. SCFA are believed to exert numerous effects on the gut, such as regulating intestinal immunity, and supplying energy for the colon epithelium. It has been reported that the SCFA butyrate can induce remission in a mouse model of UC by reducing the intestinal inflammation [9]. However, there have been few clinical studies investigating changes in the intestinal microbiota following FMT in UC patients, or the extent to which donor bacteria can colonize in the recipient. The aims of the present study were to determine whether gut dysbiosis in UC patients could be reversed by FMT, and whether pectin delays the loss of flora diversity and improves the effects of FMT.

## Methods

### Patients

This study included a single-center, randomized trial of FMT versus FMT combined with pectin treatment (FMTP) in patients with active UC admitted to the Inflammatory Bowel Disease (IBD) Treatment Center of Jinling Hospital between September 2013 and February 2015. The diagnosis of UC was made according to standard criteria. All participants provided written informed consent. The study was approved by the Institutional Ethics Committee of Jinling Hospital. Inclusion criteria were as follows: (1) age from 18 to 70 years; (2) Mayo score of 2–10 at enrollment. Exclusion criteria were as follows: (1) pregnancy or intention to become pregnant during the study period; (2) participation in any other clinical trials within the previous 3 months; (3) infectious colitis [6, 10]; (4) use of anti-TNF or methotrexate treatment within 8 weeks of inclusion; (5) use of antibiotics or probiotics within 4 weeks of inclusion. Subjects were allowed with stable doses of mesalamine or corticosteroids before inclusion [6].

### Program

Twenty patients were randomly divided into the two treatment groups (FMT and FMTP). All patients underwent FMT via colonoscopy, and the FMTP group also took oral doses of pectin (20 g/d, 50 % wt/wt) for five consecutive days following FMT. Mayo score, C-reactive protein (CRP) level, erythrocyte sedimentation rate (ESR), and Inflammatory Bowel Disease Questionnaire (IBDQ) criteria were recorded at enrollment and at weeks 4 and 12 post-treatment. Fecal samples were collected upon enrollment and at weeks 4 and 12 for bacterial spectrum analysis and fecal calprotectin analysis.

### Donor

The standardized stool donor was a healthy, unrelated adult (22-year-old female) who had not received antibiotic therapy in the 4 weeks prior to donation. The donor had no history of intestinal disease or recent gastrointestinal infection, autoimmune or other immune-mediated diseases, or malignancies. Chronic hepatitis B and C, human immunodeficiency virus, and syphilis were excluded serologically, and the donor’s stool was negative for *C. difficile*, enterohemorrhagic *Escherichia coli*, *Salmonella*, *Shigella*, *Yersinia*, *Campylobacter*, and parasites. Donor stool samples were randomly collected, and the bacterial spectrum was confirmed to be stable using 16S rRNA high-throughput analysis (Additional file 1: Figure S1).

### Donor material preparation

Aliquots (60 g) of fresh (within 6 h of defecation) fecal sample were blended with 500 ml of sterile saline for 10 min, and then filtered through three gauze pieces to remove larger sediment. The filtered fecal preparation was stored at 4 °C until FMT was performed.

### Transplantation procedure

Patients were maintained on vancomycin (500 mg orally, twice daily for three consecutive days) until 12 h prior to the FMT procedure. Polyethylene glycol electrolyte powder was used for intestinal preparation [3]. Donor material (300 ml) was administered via colonoscopy biopsy channel.

### Analysis of microbial diversity

Microbial DNA was extracted from fecal samples using an E.Z.N.A. DNA Kit (Omega Bio-tek, Norcross, GA, USA) according to the manufacturer’s protocols. The V4–V5 regions of the bacterial 16S ribosomal RNA gene were amplified by PCR (95 °C for 2 min, followed by 25 cycles of 95 °C for 30 s, 55 °C for 30 s, and 72 °C for 30 s, and a final extension of 72 °C for 5 min) using primers 515F (5′-barcode-GTGCCAGCMGCCGCGG-3′) and 907R (5′-CCGTCAATTCMTTTRAGTTT-3′), where the barcode was an eight-base sequence unique to each sample. Reactions were performed in triplicate in a 20-μl volume containing 4 μl of 5× FastPfu Buffer, 2 μl of 2.5 mM dNTPs, 0.8 μl of each primer (5 μM), 0.4 μl of FastPfu Polymerase, and 10 ng of template DNA. Amplicons were extracted from 2 % agarose gels and purified using an AxyPrep DNA Gel Extraction Kit (Axygen Biosciences, Union City, CA, USA) according to the manufacturer’s instructions, and quantified using QuantiFluor-ST (Promega, Madison, WI, USA). Purified amplicons were pooled in equimolar concentrations, and paired-end sequenced (2 × 250) on an Illumina MiSeq platform (Illumina, San Diego, CA, USA) according to standard protocols. The raw reads were deposited in the NCBI Sequence Read Archive (SRA) database.

### Fecal calprotectin (FC) test

Extract was added to 50–100 mg of stool sample until the weight:volume (g/ml) ratio reached 1:49. Following vigorous shaking, a 2-ml aliquot of this mixture was centrifuged for 5 min at 10,000 × *g*. The supernatant was used for detection of FC in accordance with the kit instructions (Buhlmann Company, Switzerland). Absorbance was measured at 450 nm. Normal levels of fecal calprotectin are <30 μg/g.

### Definition of clinical outcome

Patients were monitored for 12 weeks after treatment. Mayo scores ≤ 2 at week 12 were considered indicative of clinical remission [11–13]. A reduction in the total Mayo score of > 30 % from baseline, a 1-point improvement in tarry stools, or an increase of > 16 points in IBDQ criteria at week 12 were considered clinical responses. Two gastroenterologists specializing in IBD assessed the Mayo scores. Adverse events were documented for up to 12 weeks.

### Statistical analysis

R statistical software (version 3.1.1; http://cran.r-project.org) was used for all statistical analyses unless specified. All data are expressed as means ± SEM. Comparison within groups was performed using a Student’s *t*-test. An operational taxonomic unit (OTU) was defined using a threshold of 97 % identity by Usearch (version 7.1; http://drive5.com/uparse). A ribosomal database project classifier was used for taxonomic assignment of all sequences at the 70 % confidence level [14]. The most abundant sequence of each OTU (97 % similarity) was searched against the Silva database [15] (Release 119; http://www.arb-silva.de) using BLAST analysis to determine the phylogeny of the OTUs. Diversity was estimated using the Shannon diversity index (H’) (R package 2.7.1). Relative abundances of the OTUs at the phylum, class, order, family, genus, and species levels were calculated for each sample and used for principal component analysis (PCA), correlation analysis, and comparison analysis. The Pearson correlation coefficient (r) and Student’s *t*-test were used for correlation and comparison analysis, respectively. Significance was set at *P* < 0.05.

## Results

### Baseline characteristics of patients

Basic information on the 20 enrolled patients is shown in Table 1. All patients completed their treatment protocol, and stool samples were collected for microbiome analysis and FC test. Five stool samples were missed in the FMT group, and three in the FMTP group.

**Table 1:** Basic patient information (data are expressed as mean ± SEM)

	FMT(*n* = 10)	FMTP(*n* = 10)
Sex(male/female)	3/7	6/4
Age(years)	43.50(15)	37.40(9.92)
Drugs before reatment		
5-ASA	7	9
Prednisone	0	2(combined with 5-ASA)
Mesalazine suppository	3	1
Course of disease(years)	4.5(3)	4(2)
ESR (mm/h)	11.5(0.15)	10(6)
CRP(mg/l)	0.60(0.15)	1.76(1.51)
IBDQ	127.40(27.68)	119.25(17.88)
FC	814.15(348.69)	626.12(470.11)
Mayo score	6.60(1.12)	4.75(0.75)

### Changes in clinical outcome

**Fig. 1:**
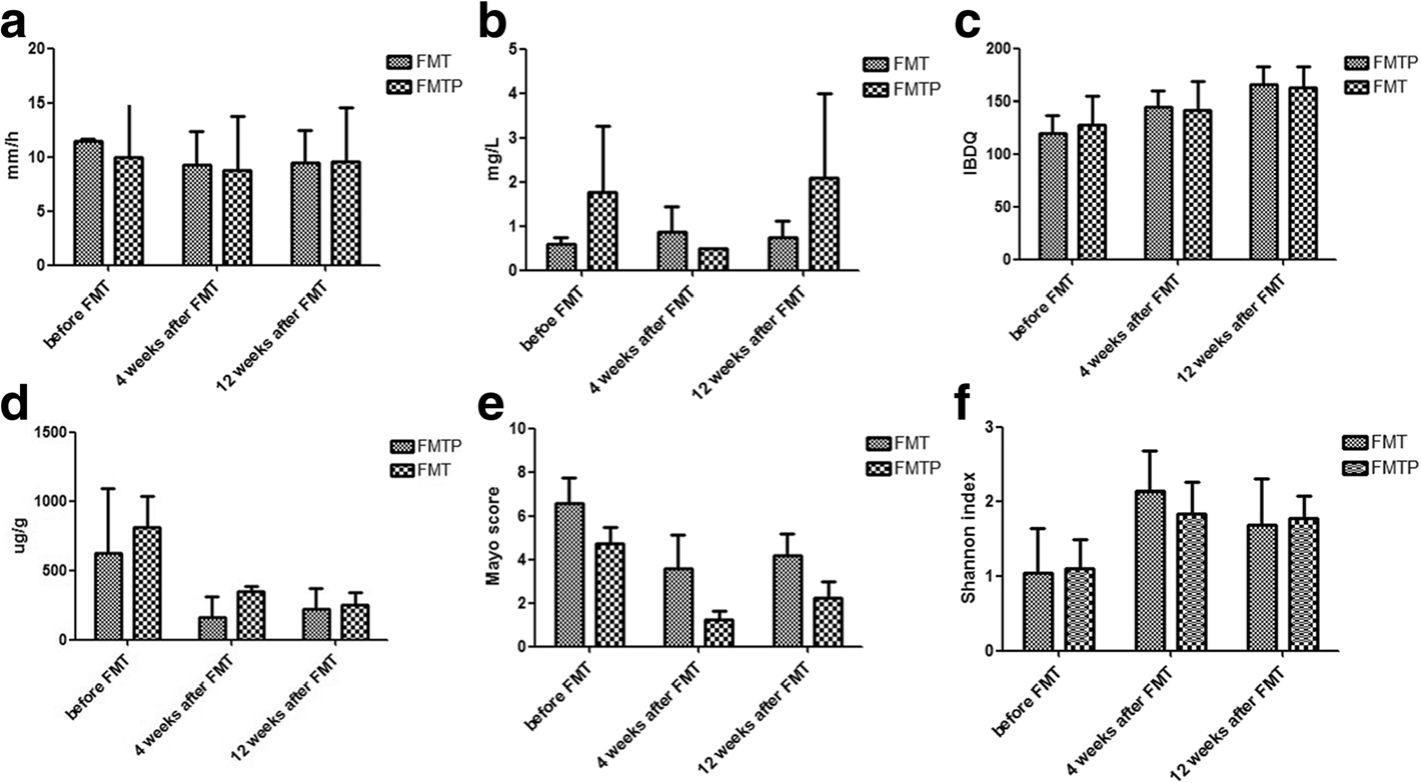
Changes in clinical indicators over time for the FMT and FMTP groups. **a**-**d** Erythrocyte sedimentation rate, C-reactive protein levels, Inflammatory Bowel Disease Questionnaire score, and fecal calprotectin levels for the two groups prior to treatment and at weeks 4 and 12 post-treatment. **e** Mayo scores at weeks 4 and week 12 post-treatment. **f** Comparison of community diversity in the two groups

There were no significant differences in CRP level, ESR, IBDQ criteria, or FC level between the two groups at weeks 4 or 12 (Fig. 1a–d). However, Mayo scores were significantly lower in the FMTP group than in the FMT group at both time points (4 weeks: 1.25 ± 0.38 vs. 3.60 ± 1.52, *P* = 0.042; 12 weeks: 2.25 ± 0.75 vs. 4.20 ± 0.96, *P* = 0.042). In addition, for both groups, the Mayo score was higher at week 12 than at week 4, indicating that the effect of FMT gradually decreased over time (Fig. 1e). At week 12, 30 % (3/10) of patients in the FMT group and 40 % (4/10) in the FMTP group achieved remission, while 70 % (7/10) and 60 % (6/10), respectively, had a clinical response. In the FMT group, three patients had a reduction in total Mayo score of > 30 %, two had a 1-point improvement in tarry stools, and four patients had increases in IBDQ criteria of > 16 points. In the FMTP group, four patients showed a reduction in Mayo score of > 30 %, four had a 1-point improvement in tarry stools, and six patients had increases in IBDQ criteria of > 16 points.

### Changes in the gut microbiota

**Fig. 2:**
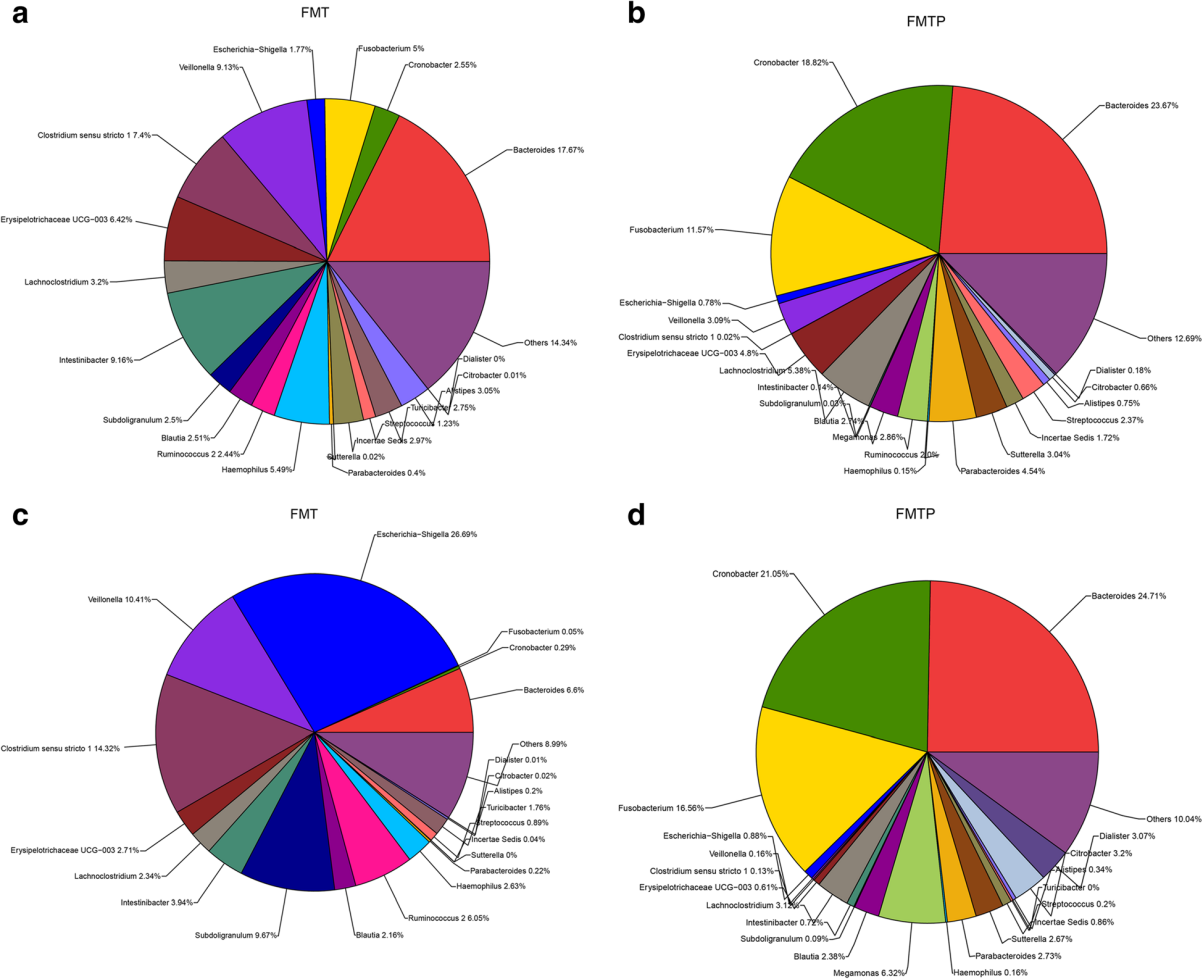
Fecal flora composition of the FMT and FMTP groups after transplantation. **a** and **b** Fecal flora composition at the genus level for the two groups 4 weeks post-transplantation. **c** and **d** Fecal flora composition at the genus level of the two groups 12 weeks post-transplantation

Eighteen samples in the FMTP group and 15 in the FMT group were analyzed using 16S rRNA-based profiling. Both treatments resulted in an increase in the Shannon diversity index compared with the baseline, indicative of greater species diversity. There was no obvious difference in fecal flora diversity (Shannon index) between the two treatment groups at weeks 4 or 12 post-transplantation (Fig. 1f, Fig. 2). To allow comparison, the Shannon index was set to 1.41 as a reference value. This was the minimum value of the diversity of the donor, and it was used to indicate how many samples had larger indexes than the reference value in either of the groups. At week 4, 77.8 % (7/9) of samples in the FMTP group and 75.0 % (6/8) in the FMT group had Shannon indexes > 1.41. At week 12, 77.8 % (7/9) of samples in the FMTP group but only 42.9 % (3/7) in the FMT group had Shannon indexes > 1.41 (Table 2), indicating that pectin helped to delay the loss of diversity in the fecal microbiota. This may be related to the lower Mayo scores observed in the FMTP group at weeks 4 and 12.

**Table 2:** Percentages of patients with a Shannon index > 1.41

Indicators	FMTP		FMT	
	Week 4	Week 12	Week 4	Week 12
Number of samples	9	9	8	7
Number of samples with Shannon index larger than 1.41	7	7	6	3
Percentage of samples with Shannon index larger than 1.41 (%)	77.78	77.78	75	42.86

Changes in the abundance of lower taxonomic ranks, such as increased numbers of Enterobacteriaceae, have been reported in patients with UC. In the current study, Shiga toxin-producing *E. coli* accounted for 16.8 % of the total gut bacterial population in UC patients prior to FMT, then decreased to 1.28 % at week 4, and returned to 13.8 % at week 12. The abundance of Shiga toxin-producing *E. coli* in the FMTP group at week 12 was only 0.88 % (Table 3).

**Table 3:** Percentages of Shiga toxin-producing *Escherichia coli* in different groups during the experimental period

	Before	4 weeks after FMT	12 weeks after FMT
FMT	16.84 %	1.77 %	26.69 %
FMTP	15.77 %	0.78 %	0.88 %

**Fig. 3:**
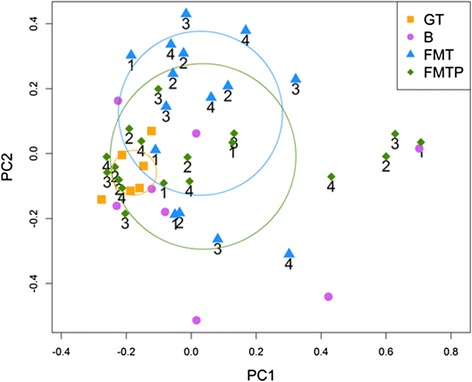
More samples closer to the donor in group FMTP than in group FMT after treatment in the PCA picture showing FMTP is more effective in maintaining the diversity of the flora

PCA based on the relative abundance of OTUs in four assigned groups (donor, before treatment, FMT, and FMTP) revealed a separation between groups on the basis of the first two principal component (PC) scores. The distances of samples in the donor group were much shorter, while samples in other groups were dispersed. To measure the distances between the samples, a circle of influence was defined for each group. The x- and y-value of the center of the circle of influence were defined as the average PC1 and PC2 values of each sample in the group. The radius was the average distance between the center and each sample in the group. There were three samples in the FMTP group within the circle of influence of the donor, and the left-most samples in the group were also closer to the circle, indicating that the composition of the microbiota of the FMTP samples was more similar to the donor than the FMT group (Fig. 3).

**Fig. 4:**
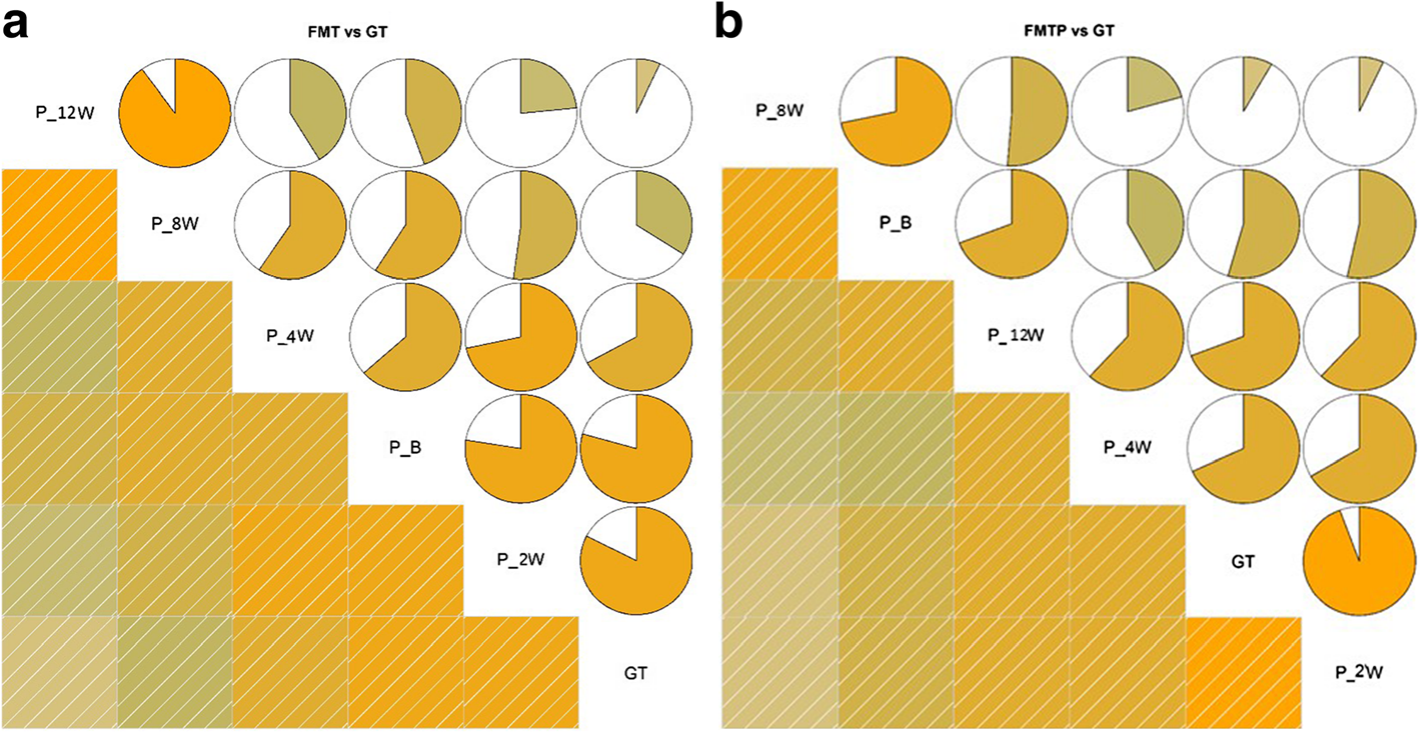
Pearson correlation coefficient (*r*) between OTU values of each sample and the donor were calculated and compared to make out whether post-treatment *r* of the sample and donor were greater than pre-treatment. 55.56 % of the post-treatment samples had larger *r* with the donor than untreated samples in FMTP group (**a**) but only 46.67 % in FMT group (**b**)

To further assess the efficiency of the two treatment protocols, we calculated the Person correlation coefficient (r) between the samples and the donor to compare the coefficients of post-treatment samples and pre-treatment samples. In the FMTP group, 55.6 % of the samples had larger correlation coefficients with the donor after treatment than untreated samples. In the FMT group, the percentage was 46.7 % (Fig. 4).

The composition of the microbiota in the FMT and FMTP groups was also compared at the phylum, class, order, family, genus, and species levels (Table 4). The differences in composition between the donor, FMTP, and FMT groups are shown in Additional file 1.

**Table 4:** Differences in the composition of gut microflora following FMT or FMTP

Level	Differences	*P*-value	Up or down^a^
Phylum	–	–	–
Class	Betaproteobacteria	0.0031	down
Order	–	–	–
Family	Acidaminococcaceae	0.0167	up
	Alcaligenaceae	0.0104	up
	Lactobacillaceae	0.0438	down
Genus	Ruminococcus	0.0257	up
	Sutterella	0.0238	down
	Turicibacter	0.0232	up
Species	Turicibacter_unclassified	0.0232	up
	Subdoligranulum_uncultured_ruminococcaceae_bacterium	0.0236	down
	Ruminococcus_torques_ATCC_27756	0.0074	up
	Phascolarctobacterium_uncultured_organism	0.0391	up
	Megamonas_uncultured_organism	0.0042	up
	Fusobacterium_varium	0.0253	up
	Fusobacterium_mortiferum	0.0475	up
Phylum	–	–	–
Class	Betaproteobacteria	0.0031	down
Order	–	–	–
Family	Acidaminococcaceae	0.0167	up
	Alcaligenaceae	0.0104	up
	Lactobacillaceae	0.0438	down
Genus	Ruminococcus	0.0257	up
	Sutterella	0.0238	down
	Turicibacter	0.0232	up
Species	Turicibacter_unclassified	0.0232	up
	Subdoligranulum_uncultured_ruminococcaceae_bacterium	0.0236	down
	Ruminococcus_torques_ATCC_27756	0.0074	up
	Phascolarctobacterium_uncultured_organism	0.0391	up
	Megamonas_uncultured_organism	0.0042	up
	Fusobacterium_varium	0.0253	up
	Fusobacterium_mortiferum	0.0475	up

^a^Relative to the FMT group

### Adverse events

The overall incidence of adverse events was 15 % (3/20). One patient showed intolerance to FMT and immediate leakage of donor material within 30 min of the procedure. Two patients developed fevers following FMT. None of the other patients experienced serious adverse events.

## Discussion

UC is a chronic inflammatory condition of the colon. It is caused by an inappropriate immune response to luminal antigens. In genetically susceptible hosts, specific changes in the composition of the intestinal microbiota can lead to activation of the mucosal immune system, resulting in chronic inflammation of the colonic mucosa [16]. Evidence from several studies has highlighted alterations in abundance and diversity of the intestinal microbiota in patients with UC.

Theoretically, introduction of probiotics may alter the dysbiosis and the aberrant immune response in UC, resulting in clinical improvement of the disease. Gut microbiota have emerged as a potential therapeutic target and a repository for novel drug discovery. Commercially available probiotics used in treatment of UC mostly include *Lactobacillus* and *Bifidobacterium* species, with the anaerobes that comprise most of the intestinal flora having been subjected to less exploration [5]. FMT, which contains the whole microbiome, was developed as a treatment to restore the commensal bacteria of patients suffering from disease-related dysbiosis.

The application of FMT for UC has been described in several publications. Previous meta-analysis showed that 63 % of patients with UC underwent remission following FMT, 76 % were able to stop taking medications, and 76 % experienced improvement in gastrointestinal symptoms [4]. However, several randomized controlled trials in 2015 showed conflicting results of FMT in UC cases [5–7]. Maintaining the diversity of flora over a long period would improve the clinical outcome and delay the recurrence of disease following FMT for treatment of UC.

SCFA, especially butyrate, have significant effects on the intestine [9, 17, 18]. Soluble dietary fiber may serve as a precursor of SCFA. No previous study has addressed whether SCFA can improve the effects of FMT in UC cases. Pectin, a polysaccharide that can be fermented into SCFA by commensal bacteria, is widely used in medicine and the food industry. The results of the current study show that pectin enhances the efficacy of FMT, as indicated by lower Mayo scores at 4 and 12 weeks in the FMTP group. Further analysis showed that pectin did not increase the diversity of the fecal microflora, but can delay the deterioration of bacterial diversity.

Differences in the short-term effects of FMT in the current study compared with those observed in previous studies might be explained by the following factors [19, 20]. Only a single infusion was administered in the current study, as it is impractical to perform repeated colonoscopies over consecutive days. In addition, many protocols recommend bowel preparation and antibiotics prior to FMT to assist in the colonization of microbiota [21]. While these steps were performed in the current study, the impact of colonic lavage and antibiotic therapy on ulcerative colitis activity is unknown [22].

Several changes in the composition of the intestinal microbiota at the phylum level have been linked to UC. These include a reduction of Firmicutes and Bacteroidetes, and an increase in Proteobacteria and Actinobacteria. In this study, the levels of Bacteroidetes increased following treatment in both the FMT and FMTP groups, but no significant changes were observed in the abundance of Firmicutes, Proteobacteria, and Actinobacteria during the study period. The composition of the intestinal microbiota was also compared at six taxonomic levels. There were no significantly different OTUs at the phylum or order levels. At class level, Betaproteobacteria were less abundant in the FMTP group. At the family level, Acidaminococcaceae and Alcaligenaceae were more abundant, while Lactobacillaceae were less abundant in the FMTP group. At the genus level, two genera, *Ruminococcus* and *Turicibacter*, were more plentiful in the FMTP group, but the abundance of *Sutterella* species was decreased. Pectin fermentation is relatively common among *Bacteroides* species, but among Gram-positive anaerobes, including Firmicutes and actinobacterial species, it is only performed by *Eubacterium eligens.* Dongowski et al. found that *Bacteroides thetaiotaomicron*, a Gram-negative anaerobe that is common in the gut, was accompanied by increased formation of SCFA from pectin. In the current study, changes in the bacterial composition were only analyzed during the bioinformatics analysis step. As such, the specific bacterial species composition associated with pectin degradation was not determined. However, this is something we will continue to explore, and we hope to focus future studies on identifying which bacterial genera are involved in pectin degradation and improvement of Mayo scores in UC patients.

As FMT as a therapeutic approach has evolved, ethical issues around the public aesthetic and access to medical technology have become a focal point. The fact that the treatment involves the transfer of fecal matter greatly reduces its acceptance; however, research indicates that most UC patients would accept FMT to alleviate symptoms and improve quality of life. As such, aesthetic requirements are not a high priority. Researchers are constantly trying to improve FMT, including the development of FMT capsules, which would further reduce the visual and olfactory impediments. Another problem is medical technology standardization issues. A consensus needs to be reached regarding bowel preparation prior to FMT, selection criteria for the donor and recipient, and the route of transplantation. Therefore, further multi-center randomized controlled trials and cooperation between research groups are needed to develop a standard and effective FMT procedure. This study has several limitations, including the small number of subjects and that patients with severe UC were excluded. Drug therapy before and after FMT may also influence the results. Further trials enrolling a larger number of patients, and allowing patients to stop medication to observe the effect of FMT on UC, are needed. However, the current study confirmed that pectin delayed the loss of diversity of transplanted gut flora, and enhanced the effects of FMT in UC cases.

## Conclusion

FMT has a short-time effect for mild to moderate UC and pectin further decreased the Mayo score by preserving the diversity of the gut flora following FMT for UC and enhanced the effect of FMT.

## Additional file

Additional file 1: Figure S1. Community structure of random donor stool samples. (TIF 103 kb)

## Abbreviations

FC: Fecal calprotectin
FMT: Fecal microbiota transplantation
IBDQ: Inflammatory Bowel Disease Questionnaire
OTU: Operational taxonomic unit
PC: Principal component
SCFA: Short-chain fatty acid
UC: Ulcerative colitis
