# Supplementary figures and images for: Pectin enhances the effect of fecal microbiota transplantation in ulcerative colitis by delaying the loss of diversity of gut flora
Source: BMC Microbiol. 2016 Nov 3;16:255. doi: 10.1186/s12866-016-0869-2 (PMC5095982; doi:10.1186/s12866-016-0869-2)

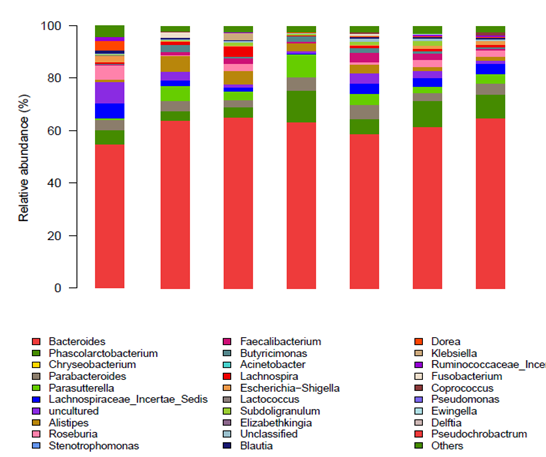

Supplement: Additional file 1: Figure S1. — Community structure of random donor stool samples. (TIF 103 kb) [file 12866_2016_869_MOESM1_ESM.tif]
